# Food additives: Assessing the impact of exposure to permitted emulsifiers on bowel and metabolic health – introducing the FADiets study

**DOI:** 10.1111/nbu.12408

**Published:** 2019-11-25

**Authors:** D. Partridge, K. A. Lloyd, J. M. Rhodes, A. W. Walker, A. M. Johnstone, B. J. Campbell

**Affiliations:** ^1^ The Rowett Institute University of Aberdeen Aberdeen UK; ^2^ Institute of Translational Medicine University of Liverpool Liverpool UK

**Keywords:** bacterial translocation, emulsifiers, food additives, gut microbiota, intestinal health and inflammation, metabolic syndrome

## Abstract

Emulsifiers are common components of processed foods consumed as part of a Western diet. Emerging *in vitro* cell‐line culture, mouse model and human intestinal tissue explant studies have all suggested that very low concentrations of the food emulsifier polysorbate 80 may cause bacterial translocation across the intestinal epithelium, intestinal inflammation and metabolic syndrome. This raises the possibility that dietary emulsifiers might be factors in conditions such as coronary artery disease, type 2 diabetes and Crohn's disease. The potential mechanism behind the observed effects of this emulsifier is uncertain but may be mediated via changes in the gut microbiota or by increased bacterial translocation, or both. It is also unknown whether these effects are generalisable across all emulsifiers and detergents, including perhaps the natural emulsifier lecithin or even conjugated bile acids, particularly if the latter escape reabsorption and pass through to the distal ileum or colon. A major objective of the Medical Research Council (MRC)‐funded *Mechanistic Nutrition in Health* (*MECNUT*) *Emulsifier* project is therefore to investigate the underlying mechanisms and effects of a range of synthetic and natural emulsifiers and detergents *in vitro* and *in vivo*, and to determine the effects of a commonly consumed emulsifier (soya lecithin) on gut and metabolic health through a controlled dietary intervention study in healthy human volunteers – the *FADiets* study. This report provides an overview of the relevant literature, discussing the impact of emulsifiers and other additives on intestinal and metabolic health, and gives an overview of the studies being undertaken as part of the *MECNUT Emulsifier* project.

## Introduction

### Permitted food additives in the Western diet

Food additives are substances which are purposely added to food and drink products to perform certain functions, such as to colour, sweeten and/or stabilise and preserve (Mepham 2011). The use of food additives has increased dramatically since they were intentionally used for food preservation in the early 1800s (Fennema 1987; Carocho *et al.* 2014; Zinöcker & Lindseth 2018). Today, when grocery shopping, it is nearly impossible to avoid processed foods, particularly in the consumption of a typical Western diet – a modern dietary pattern that is characterised by low intake of fruit, legume and vegetable fibre and high intake of red meat, dairy, eggs and refined grains, saturated fat, sugar and salt along with increased exposure to additives due to their use in processed foods (Slimani *et al.* 2009; Adams & White 2015; Zhong *et al.* 2018). Some processed foods can form part of a healthy, balanced diet (*e.g.* wholemeal bread; low‐sugar, high‐fibre breakfast cereals), whilst others may be considered more detrimental for health (*e.g.* processed meats, high‐fat dairy and bakery products, confectionery, foodstuffs containing hydrogenated oils and high fructose corn syrups) (Carocho *et al.* 2014; Zinöcker & Lindseth 2018). Today, there are over 2500 permitted additives that are included in foods to enhance appearance, smell, texture and taste, and/or to extend shelf‐life (Branen *et al.* 2001; Carocho *et al.* 2014). In the European Union, these are classified into 26 functional classes (Table 1), with some of the most commonly consumed additives being sweeteners, colourants and emulsifiers/surfactants (Huvaere *et al.* 2012; Roberts *et al.* 2013; Stevens *et al.* 2014). Data acquired through surveys from a number of populations (including the UK, mainland Europe, the US, Canada, New Zealand and South America) have suggested consumption of additive‐containing processed food products can contribute to between 25 and 50% of total daily energy intake (Slimani *et al.* 2009; Moubarac *et al.* 2013; Adams & White 2015; Costa Louzada *et al.* 2015; Steele *et al.* 2016; Zhong *et al.* 2018; Cediel *et al.* 2018), whilst total intake of food additives per person in industrial countries has been suggested to be 7‐8 kg per annum (Mepham 2011), although this represents only 0.7‐0.8% of the total food intake of a US adult consuming ~ 996 kg per annum (National Geographic/FAOSTAT 2011).

**Table 1:** Functional classes and examples of additives in foods – adapted from European Parliament (2008)

Functional class	Description	Example additive (E number*)
Acidity regulators	Alter or control the acidity or alkalinity pH of a foodstuff	E325 Sodium lactate
Acids	Increase the acidity of a foodstuff and/or impart a sour taste to it	E507 Hydrochloric acid
Anti‐caking agents	Reduce the tendency of individual particles of a foodstuff to adhere to one another	E341 Calcium phosphate
Anti‐foaming agents	Prevent or reduce foaming	E905a Mineral oil
Antioxidants	Prolong the shelf‐life of foods by protecting them against deterioration caused by oxidation, such as fat rancidity and colour changes	E300 Ascorbic Acid
Bulking agents	Contribute to the volume of a foodstuff without contributing significantly to its available energy value	E336 Potassium tartrates
Carriers	Dissolve, dilute, disperse or otherwise physically modify a food additive or a flavouring, food enzyme, nutrient and/or other substance added for nutritional or physiological purposes to a food without altering its function (and without exerting any technological effect themselves) to facilitate its handling, application or use	E1200 Polydextrose
Colours	Add or restore colour in a food, and include natural constituents of foods and natural sources, which are normally not consumed as foods as such and not normally used as characteristic ingredients of food	E100 Curcumin
Emulsifiers	Make it possible to form or maintain a homogenous mixture of two or more immiscible phases such as oil and water in a foodstuff	E322 Lecithin
Emulsifying salts	Convert proteins contained in cheese into a dispersed form and thereby bring about homogenous distribution of fat and other components	E325 Sodium lactate
Firming agents	Make or keep tissues of fruit or vegetables firm or crisp, or interact with gelling agents to produce or strengthen a gel	E333 Calcium citrates
Flavour enhancers	Enhance the existing taste and/or odour of a foodstuff	E620 Glutamic acid
Flour treatment agents	Added to flour or dough to improve its baking quality	E927b Carbamide
Foaming agents	Make it possible to form a homogenous dispersion of a gaseous phase in a liquid or solid foodstuff	E999 Quillaia extract
Gelling agents	Give a foodstuff texture through formation of a gel	E441 Gelatine
Glazing agents	When applied to the external surface of a foodstuff, impart a shiny appearance or provide a protective coating	E901 Bees wax
Humectants	Prevent foods from drying out by counteracting the effect of an atmosphere having a low degree of humidity, or promote the dissolution of a powder in an aqueous medium	E965 Maltitol
Modified starches	Obtained by one or more chemical treatments of edible starches, which may have undergone a physical or enzymatic treatment, and may be acid or alkali thinned or bleached	E1404 Oxidised starch
Packaging gases	Gases other than air, introduced into a container before, during or after the placing of a foodstuff in that container	E938 Argon
Preservatives	Prolong the shelf‐life of foods by protecting them against deterioration caused by micro‐organisms and/or which protect against growth of pathogenic micro‐organisms	E200 Sorbic acid
Propellants	Gases other than air that expel a foodstuff from a container	E942 Nitrous oxide
Raising agents	Substances or combinations of substances that liberate gas and thereby increase the volume of a dough or a batter	E500 Sodium carbonate
Sequestrants	Form chemical complexes with metallic ions	E385 Calcium disodium ethylene diamine tetraacetate
Stabilisers	Make it possible to maintain the physico‐chemical state of a foodstuff	E415 Xanthan gum
Sweeteners	Impart a sweet taste to foods or in table‐top sweeteners	E955 Sucralose
Thickeners	Increase the viscosity of a foodstuff	E1400 Dextrin

* FSA (2018a).

There have been reports of associations between ‘ultra‐processed’ foods and adverse health outcomes in populations around the world, including allergic and autoimmune disorders, some types of cancer, cardiovascular diseases and metabolic disorders, such as type 2 diabetes and obesity (Csáki 2011; Fardet 2018; Srour *et al.* 2019). ‘Ultra‐processed’ foods have been defined by researchers in South America as industrial formulations ‘…made from processed substances extracted or refined from whole foods… are typically energy dense; have a high glycaemic load; are low in dietary fibre, micronutrients, and phytochemicals; and are high in unhealthy types of dietary fat, free sugars, and sodium’ (Monteiro *et al.* 2013) and ‘formulations made mostly or entirely from substances derived from foods and additives, with little if any intact…food’ (Monteiro *et al.* 2017). Furthermore, the researchers state that ‘intense palatability’ achieved by high content of fat, sugar, salt and cosmetic and other additives (along with other factors such as marketing) encourages overconsumption of such foods (Monteiro *et al.* 2013) and that ‘classes of additives found only in ‘ultra‐processed’ products include those used to imitate or enhance the sensory qualities of foods or to disguise unpalatable aspects of the final product. These additives include dyes and other colours, colour stabilizers; flavours, flavour enhancers, non‐sugar sweeteners; and processing aids such as carbonating, firming, bulking and anti‐bulking, de‐foaming, anti‐caking and glazing agents, emulsifiers, sequestrants and humectants (Monteiro *et al.* 2017). Suggested examples of ‘ultra‐processed’ foods include ice creams, cake mix, powered soups, reconstituted meat products, packaged ‘instant’ noodles and pre‐prepared meat, fish, vegetable and cheese dishes (Monteiro *et al.* 2013; Monteiro *et al.* 2017). It should be noted, however, that the definition of ‘ultra‐processed’ foods, and indeed examples of foods within this category, is highly variable and open to interpretation, highlighting that foods are perhaps better categorised based on their nutrient value rather than level of processing and presence of particular ingredients including additives (Gibney 2018). Regulatory bodies ensure that food additives are rigorously tested for safety and additives continue to undergo long‐term monitoring for their effects on chronic health conditions. Food additives that pass these safety tests are given an ‘E’ number which must be listed on packaging (FSA 2018b).

### Widespread use of emulsifiers in the Western diet

Whilst consumption of some food additives (*e.g.* artificial sweeteners such as sucralose) can be limited through food choice, it may be much more difficult to avoid ingestion of emulsifiers (also known as surfactants or detergents) because they are commonly added to a wide variety of foods within the modern Western diet (see Table 2). Whilst regulatory bodies can define limits on amounts that can be added to food products, information regarding actual content within foods is lacking on food labels, limiting our knowledge of levels consumed and our ability to avoid consumption of a large, diverse array of surfactant compounds used in foods (Halmos *et al.* 2019). The term ‘emulsifier’ is commonly used for surfactants that are used in both the food and pharmaceutical industries, whilst the term ‘detergent’ is more commonly used to refer to specific surfactants used in household and cleaning products (*e.g*. washing liquids, shampoos, toothpastes). A wide range of surfactants is available, both those that are man‐made (*e.g.* polysorbates, derived from polyethoxylated sorbitan and oleic acid, also known as Tween) and natural (*e.g*. lecithin), many of which can also be modified chemically to alter their properties (Table 2). Surfactants have the common property of being amphophilic [*i.e.* with a molecular structure that includes both a hydrophile (water‐loving, polar) and a lipophile (fat‐loving) component]. Lipophilic components tend to be similar, but hydrophilic components vary and form the basis for the classification of surfactants as non‐ionic, anionic, cationic and amphoteric. Within the food industry, synthetic non‐ionic polysorbates were introduced in the 1930s, initially incorporated into margarines and then used extensively in the baking industry as preservatives to prevent staling, and enhance firmness and volume of bakery goods (Langhans & Thalheimer 1971; Hasenhuettl & Hartel 2008). Polysorbates, and other synthetic emulsifiers, are frequently incorporated into dietary products, either singly or in combination, usually at doses of 0.2‐0.5% of flour weight (Csáki 2011). Blended with other emulsifiers, such as natural and synthesised sources of mono‐ and diglycerides, polysorbates aid the formation of stable oil‐in‐water emulsions needed for margarines, sauces and dressings, to hold the fat in ice creams and to retard fat bloom (separation of cocoa butter) in chocolate products. In many cases, the same synthetic emulsifiers are used in pharmaceutical products as absorption enhancers (Hasenhuettl & Hartel 2008). Data on the gastrointestinal fate of many emulsifiers are not readily available, although a recent review has highlighted the likely metabolic process for some key surfactants and thickening agents (Halmos *et al.* 2019). Natural emulsifiers such as lecithin (phosphatidylcholine) are broken down to choline‐rich nutrients on passage through the small intestine by intestinal lipases (Szuhaj 1989; JECFA 1974a) and then acted upon by bacteria to produce triethylamine (Tang *et al.* 2013). There is a greater resistance to breakdown by digestion of synthetic emulsifiers, such as the polysorbate series of surfactants, as seen for polysorbate 80 where the fatty acid moieties are effectively metabolised but the sorbitol part of the molecule is seen to be highly resistant to digestion in the intestine (JECFA 1974b; Singh *et al.* 2009). Likewise, carboxymethylcellulose is a non‐digestible polysaccharide polymer, hence its common use as a thickening agent and stabilizer in food emulsions (Halmos *et al.* 2019). Citric acid esters of mono‐ and diglycerides used to stabilise emulsions in food and infant formulas were thought be completely hydrolysed in the gut into constituent free fatty acids, glycerol and citric acid, and however, recent evidence suggests that the ester bond between citric acid and glycerol is likely not fully hydrolysable (Amara *et al.* 2014). More work needs to be undertaken in this area.

**Table 2:** Common emulsifiers (compound name, ‘E’ number, food and other uses)

Category	Compound	‘E’ number*	Food uses	Other uses
1. Synthesised emulsifiers	Mono‐ and diglycerides of fatty acids (glyceryl monostearate, glyceryl distearate)	E471	Bread, cakes, desserts, margarines, spreads, ice cream, chewing gum	
	Diacetyl tartaric acid ester of mono‐ and diglycerides (DATEM)	E472e	Baked goods, beverage whiteners, cream, chewing gum, processed meat and poultry, sauces, coffee	
	Sodium stearoyl‐2‐lactylate	E481	Bread, cake, flour products, ice cream, coffee, soft drinks, creams, cookies, crackers, pasta	
	Sucrose esters of fatty acids: Sucrose monolaurate Sucrose monodecanoate Sucrose octaacetate	E473	Sauces, salad dressings, candy, chocolate, baked goods, icing and fillings, ice cream, soft drinks, cream, baby food, soups, chewing gum, desserts	Animal feed, pharmaceutical tablets
	Polysorbates (Tweens): Polyoxyethene sorbitan monolaurate (PS20) Polyoxyethene sorbitan monooleate (PS80) Polyoxyethene sorbitan monopalmitate (PS40) Polyoxyethene sorbitan monostearate (PS60) Polyoxyethene (20) sorbitan tristearate (PS65)	E432 E433 E434 E435 E436	Whipped toppings, salad dressings, cakes, cake mixes, edible oils, cake icings and filling, dairy product substitutes, chocolate syrups, ice creams, coffee	Cosmetics, shampoos, floor cleaner, vaccines, drug formulations
	Sorbitans: Sorbitan monostearate (Span 60) Sorbitan tristearate (Span 65) Sorbitan monolaurate (Span 20) Sorbitan monooleate (Span 80) Sorbitan monopalmitate (Span 40) Sorbitan trioleate (Span 85)	E491 E492 E493 E494 E495 E496	Bread, baked goods, active dry yeast, beverages, dairy products, non‐dairy alternatives, margarine and spreads, chocolate, confectionary	Leather brighteners, plastics, synthetics, creams, pesticides, cosmetics
	Methylcellulose	E461	Ice cream, nutritional supplements, vegetarian products, restructured seafood, batters	Shampoos, toothpaste, lubricant, treatment for constipation, construction materials, glue, paper and textiles, movie special effects
2. Natural emulsifiers	Lecithin: Egg yolk Soya (powder/granules) Sunflower	E322	Ice cream, margarine, candy, chocolate, soft and hard caramels, chewing gum, cakes, pastries, bread, biscuits, baby formula, hot chocolate, protein powder	Drug formulations (particularly IV), moisturisers, lipstick, foundations, shampoo, soap, creams and lotions, dying leather and textiles, paints and inks, biocides, animal feed
	Sucrose acetate isobutyrate	E444	Cocktail mixers, beer, malt beverages	Cosmetics/skin care, fragrance fixative, hair care/styling products
3. Household detergents	Dodecyltrimethylammonium bromide (DDTMA)			Paint strippers, bactericidal lotions, antiseptics, soaps, purifying or cleansing agents
	Sodium dodecyl sulphate/sodium lauryl sulphate (SDS)	E487	Whipped products	Household and car cleaning products, laxatives and drug formulation, topical microbicide

* FSA (2018a). IV, intravenous.

### Potential concerns regarding the use of emulsifiers in the Western diet

Emerging evidence suggests that permitted dietary emulsifiers may impact on gut health through impairing intestinal barrier function, thus increasing antigen exposure, and/or by modulating the microbiota, thus potentially increasing the incidence of inflammatory bowel disease (IBD) and metabolic syndrome (Roberts *et al.* 2010; Csáki 2011; Chassaing *et al.* 2015; Cani & Everard 2015). We have highlighted significant correlations between emulsifier consumption per capita and Crohn’s disease incidence across countries/continents, particularly in Japan, where there has been a particularly marked recent increase in Crohn’s disease (Roberts *et al.* 2013). Other key food stabilisers and additives, including maltodextrin, have been associated with increased early life intestinal stress, damage and inflammation in animal studies (Arnold & Chassaing 2019). For example, mice consuming a maltodextrin‐rich diet (5% w/v in drinking water over a period of 45 days) displayed an increased susceptibility to intestinal damage and endoplasmic reticulum stress (where improper folding and secretion of intestinal epithelial cell proteins leads to impairment of the intestinal barrier and activation of inflammatory responses in the host) (Laudisi *et al.* 2019). Likewise, in mice, ingestion of drinking water containing the emulsifier/thickener carboxymethylcellulose (a 2% w/v solution for 3 weeks) induced changes to their intestinal structure and promoted leukocyte migration to the intestinal lumen (Swidsinski *et al.* 2009). Exposure to carboxymethylcellulose in this study though [∼66 mg/kg bodyweight/day based on a 30g mouse (Vo *et al.* 2019)] is 2‐3 times higher than the estimated mean daily exposure seen in the US population (Shah *et al.* 2017). Potential effects of food additives on the gut microbiome have generally been overlooked; however, emerging evidence, mainly from animal studies, suggests that several common food additives, not just emulsifiers, can induce microbiota‐mediated adverse effects (see Table 3). Taken together, the emerging effects on intestinal inflammation and gut microbiota are consistent with those observed in IBD. Food exclusion diets for Crohn’s disease, which encourage the avoidance of additive‐rich ‘processed foods’, have been observed to induce remission, although lots of other dietary factors may be involved (Sigall‐Boneh *et al.* 2014; Lee *et al.* 2018).

**Table 3:** Common food additives present in a Western diet and their suggested impact on the gut microbiota and/or host physiology (not an exhaustive list)

Type of additive	Additive and dose	Model	Effect on microbiota	Effect on host physiology	Reference
Colour	Titanium dioxide (2.3 x 10^5^–2.3 x 10^9^ particles/ml)	Human colon cells	Not determined	Decrease in absorptive microvilli, decreased nutrient uptake	Guo *et al. *(2017)
Emulsifier	Carboxymethylcellulose (2% w/v within drinking water for 3 weeks)	Mice (*Il10^−/−^*)	Bacterial overgrowth	Intestinal (small bowel) inflammation	Swidsinski *et al. *(2009)
Emulsifier	Carboxymethylcellulose, or Polysorbate 80 (0.1 to 1% v/v within drinking water for 12 weeks)	Mice (*Il10^−/−^*, *Tlr5^−/−^* & C57BL/6)	Microbiota encroachment, altered species composition, increased pro‐inflammatory potential	Colitis, metabolic syndrome	Chassaing *et al. *(2015)
Emulsifier	Carboxymethylcellulose, or Polysorbate 80 (0.1 to 1% v/v faecal suspension culture)	M‐SHIME human colon model	Not determined	Increased levels of bioactive flagellin (increased pro‐inflammatory potential)	Chassaing *et al. *(2017)
Emulsifier	Polysorbate 80 (1% v/v per kg bodyweight via gavage, daily for 4 weeks)	Mice (C57BL/6)	Altered microbiota composition	Intestinal inflammation, obesity, impaired glycaemic tolerance, liver dysfunction	Singh *et al. *(2016)
Emulsifier	Polysorbate 80 (1% w/v in drinking water for 8 weeks)	Mice (C57BL/6J)	Altered microbiota composition	Enhanced indomethacin‐induced intestinal damage	Furuhashi *et al. *(2019)
Emulsifier	Glycerol monolaurate (basal diet supplemented with 150 mg/kg ingested daily for 8 weeks)	Mice (C57BL/6)	Altered microbiota composition	Metabolic syndrome, systemic low‐grade inflammation	Jiang *et al. *(2018)
Emulsifier	Methylcellulose (150 g/kg in chow for 7 days)	Mice (*Rag1^‐/‐^* & C57BL/6J)	Not determined	Increased severity of colitis	Llewellyn *et al. *(2018)
Preservatives	Silver nanoparticles (0, 11.4, 114 and 1140 μg Ag NP/kg bodyweight/day for 28 days)	Mice (C57BL/6)	Altered microbiota composition	Not determined	Van Den Brûle *et al. *(2016)
Sweetener	Sucralose (by oral gavage 100, 300, 500, or 1000 mg/kg/day for 12 weeks)	Rats (Sprague Dawley)	Altered microbiota composition	Not determined	Abou‐Donia *et al. *(2008)
Sweetener	Sucralose (0.1 mg/ml within drinking water for 6 months)	Mice (C57BL/6J)	Altered microbiota composition	Altered bile acids, elevated pro‐inflammatory gene expression in the liver	Bian *et al. *(2017b)
Sweetener	Sucralose (1.08, 3.5 and 35 mg/ml within drinking water for 6 weeks)	Mice (SAMP, AKR, and C57BL/6J)	Altered microbiota composition	Increased ileal tissue myeloperoxidase activity	Rodriguez‐Palacios, *et al. *(2018)
Sweetener	Saccharin (0.1 mg/ml within drinking water for 5 weeks)	Mice (C57BL/6) and humans	Altered microbiota composition (mice only, humans not studied)	Glucose intolerance (mice and humans)	Suez *et al. *(2014)
Sweetener	Saccharin (0.3 mg/ml within drinking water for 6 months)	Mice (C57BL/6J)	Altered microbiota composition	Liver inflammation	Bian *et al. *(2017c)
Sweetener	Aspartame (5–7 mg/kg/day for 10 weeks)	Rats (WT)	Altered microbiota composition	Glucose intolerance	Palmnäs *et al. *(2014)
Sweetener	Acesulfame K (37.5 mg/kg/day for 4 weeks)	Mice (CD‐1)	Altered microbiota composition	Weight gain (male mice only)	Bian *et al. *(2017a)
Thickener	Maltodextrin (1 to 5% w/v within drinking water over a period of 45 days)	Mice (Balb/c)	No effect on microbiota composition	Altered mucus barrier, increased intestinal inflammation	Laudisi *et al. *(2019)

Adapted from Zinöcker and Lindseth (2018). Ag NP, silver nanoparticles.

### Importance of intestinal microbiota in the pathogenesis of human disease

Recent advances in next‐generation sequencing technology have allowed for a greater expansion in our knowledge of the gut microbiota (Simpson & Campbell 2015; Malla *et al.* 2018). The human gut microbiota, established early in life and becoming stable by around 2‐3 years of age, is influenced by numerous factors including diet, exposure to antibiotics, inflammation and exercise throughout the life course (Arrieta *et al.* 2014). Perturbations in microbiota composition and activity have been associated with inflammation and with various other conditions including obesity, metabolic syndrome (associated with the risk of developing cardiovascular disease and type 2 diabetes), IBD and colorectal cancer (CRC) (Arrieta *et al.* 2014; Simpson & Campbell 2015).

Broadly, similar differences in microbiota composition are commonly observed in the faeces, and more importantly, in the mucosa‐associated intestinal microbiota which promotes low‐grade inflammation in both IBD and metabolic syndrome (Everard & Cani 2013; Schaubeck & Haller 2015; Michalak *et al.* 2016). In IBD, common changes include a reduction in key Gram‐positive bacteria from within the phylum *Firmicutes* and an increase in Gram‐negative *Proteobacteria*, especially *Enterobacteriaceae* such as *Escherichia coli* pathovars associated with patient bowel lesions and which have been demonstrated to induce intestinal inflammation and inflammation‐associated CRC in mice (Arthur *et al.* 2012; Merga *et al.* 2014). In a mouse model of metabolic syndrome, high‐fat diet‐induced diabetes is preceded by an increase in mucosa‐associated *Enterobacteriaceae*, including *E. coli* that are actively translocated into mesenteric fat and to the blood (Amar *et al.* 2011). Human studies have since confirmed that faecal transplantation from lean donors can improve the insulin sensitivity of the recipients, supporting the role of intestinal microbiota composition as a contributor to the development of metabolic syndrome (Vrieze *et al.* 2012; Nieuwdorp *et al.* 2014). We, and others, have reported an increase in mucosally associated *E. coli* in both Crohn’s disease and CRC (Swidsinski *et al.* 1998; Darfeuille‐Michaud *et al.* 2004; Martin *et al.* 2004). M (microfold) cells overlying ileal Peyer’s patches and smaller lymphoid follicles in the colon are the likely portal of entry for Crohn’s disease *E. coli* (Chassaing *et al.* 2011; Dogan *et al.* 2014; Prorok‐Hamon *et al.* 2014).

### What are the potential mechanisms behind the effects of synthetic food emulsifiers on health and are they generalisable to all emulsifiers?

**Figure 1:**
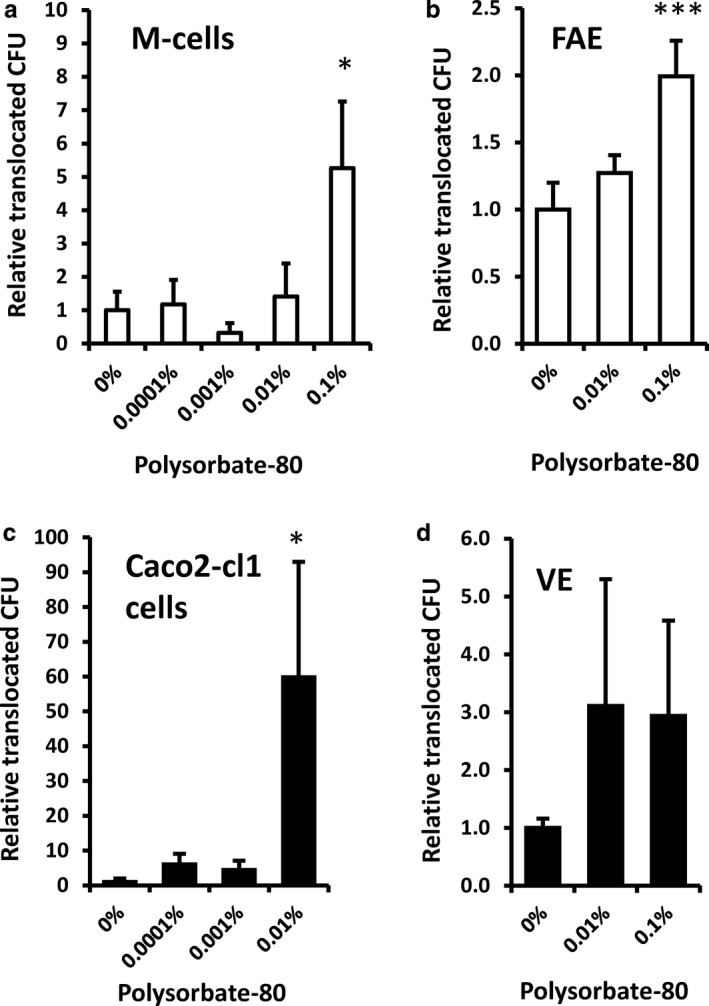
Dietary emulsifier polysorbate 80 increases translocation of *E. coli* across intestinal epithelial cell cultures (a and c) and intestinal ileum epithelium mounted in Ussing chambers (b and d). M (microfold)‐cell (Caco2‐cl1/Raji B cell co‐culture) model (a), Caco2‐cl1 intestinal cell monolayers (c), human ileal villous epithelium (VE) (d) or follicle‐associated epithelium (FAE) overlying Peyer’s patches (b). *, *p* < 0.05; **, *p* < 0.01; Kruskal–Wallis analysis of variance (ANOVA) corrected for multiple comparisons; n = 4‐8). Reproduced from Roberts *et al. *(2010) with permission from BMJ Publishing Group Ltd – Copyright clearance center Licence number 4638800129868. CFU, colony‐forming unit

Remarkably, there has been little study of the potential harmful effects of ingested detergents or emulsifiers in humans. Whilst investigating the impact of dietary components on bacterial–epithelial interactions relevant to IBD and CRC, we explored the hypothesis that increases in intestinal epithelial barrier permeability to bacteria might result from ingestion of emulsifiers or detergents (Roberts *et al.* 2010). We showed that the presence of permitted food emulsifier polysorbate 80, at low concentrations (0.01‐0.1% v/v) that might plausibly be present in the distal ileum of someone consuming a Western‐style diet, markedly increased translocation of mucosa‐associated *E. coli* across epithelial cell monolayers and across human ileal mucosa explants cultured in Ussing chambers (Roberts *et al.* 2010). This occurred across both M cells and across villous epithelium which would not normally allow entry of bacteria in healthy individuals (Fig. 1). At these low concentrations, bacterial translocation was transcellular (*i.e.* through cells) and not via the paracellular route (*i.e.* through increased ‘leakiness’ of intercellular tight junctions).

**Figure 2:**
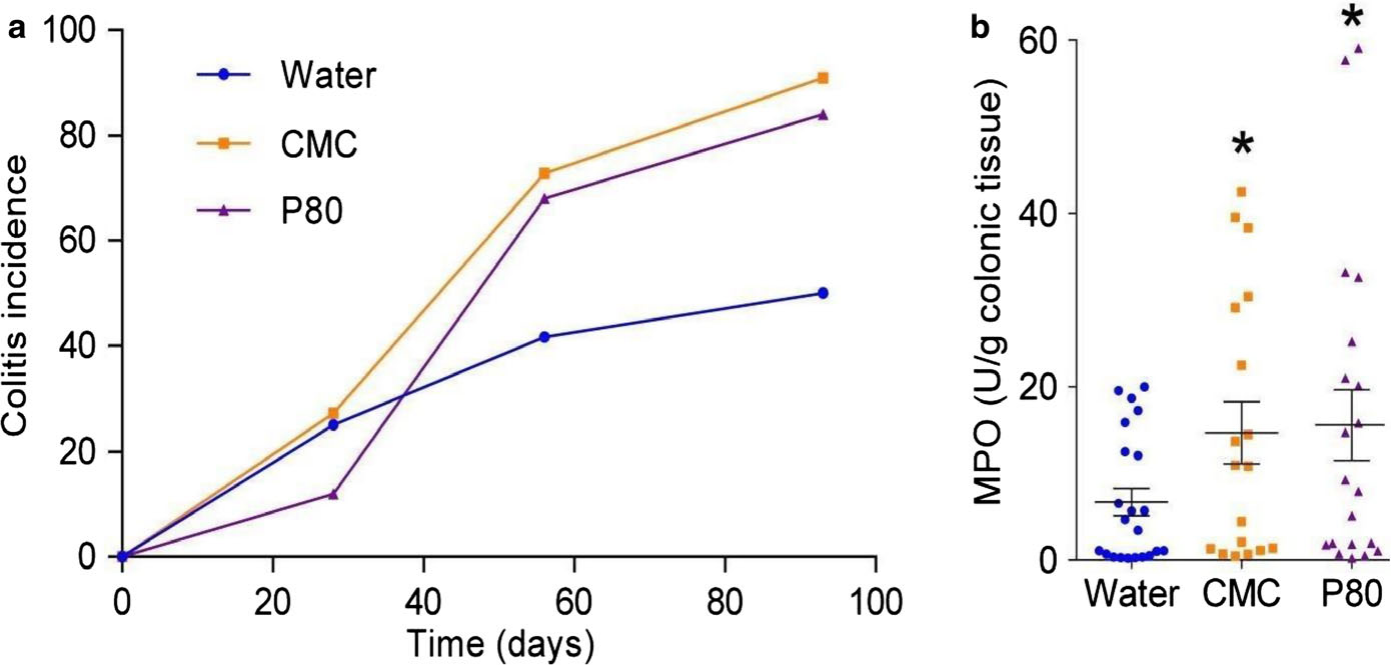
Emulsifiers polysorbate 80 (P80) and carboxymethylcellulose (CMC) administered to drinking water (1.0% v/v for 12 weeks) promote colitis (incidence of epithelial damage and inflammatory infiltrate, as determined by histology, in colonic tissues over time) (a) and increase colonic tissue myeloperoxidase (MPO) (b) in *Il10^‐/‐^* mice, and low‐grade intestinal inflammation in wild‐type mice (not shown). Points are from individual mice. *p < 0.05 compared to water‐treated group, using one‐way ANOVA corrected for multiple comparisons. Reproduced from Chassaing *et al. *(2015) with permission from Springer Nature – Copyright clearance center Licence number 4638801338133 [Colour figure can be viewed at http://www.wileyonlinelibrary.com/]

Benoit Chassaing and colleagues undertook *in vivo* animal studies where ingestion by mice of polysorbate 80 at higher concentrations [up to 1% v/v in their drinking water for 13 weeks; about equivalent to 2500 mg/kg bodyweight/day (see Vo *et al.* 2019)] caused depletion of the mucus barrier, allowing for closer apposition between luminal bacteria and the intestinal epithelium. More severe inflammation was observed in colitis‐susceptible interleukin‐10 knockout (*Il10^‐/‐^*) mice (Chassaing *et al.* 2015). Although ingestion of emulsifiers did not alter the total bacterial load in the faeces, it did significantly increase the number of bacteria adherent to the colon in both wild‐type and *Il10^‐/‐^* mice and altered the overall composition of the microbiota, including increasing the predominance of potentially inflammation‐promoting *Proteobacteria*. Intriguingly, they also found that mice fed polysorbate 80 developed low‐level inflammation and metabolic syndrome (Fig. 2). These changes, including a weakened mucus layer, were not seen in germ‐free mice lacking a microbiota and but were observed upon transfer of faeces from emulsifier‐fed mice to germ‐free recipients. Similar effects were seen in mice consuming diets containing carboxymethylcellulose (Chassaing *et al.* 2015), arguably a food thickener rather than a true emulsifier, but which has been shown previously in other mouse studies to induce inflammation in the small intestine (Swidsinski *et al.* 2009). Subsequent mouse studies showed that both polysorbate 80 and carboxymethylcellulose also potentiated intestinal inflammation associated with CRC (Viennois *et al.* 2017).

Chassaing and colleagues also recently showed that emulsifiers can cause striking changes in the microbiota. When added to a dynamic *in vitro* slurry that mimicked a human colonic microbial culture, both polysorbate 80 and carboxymethylcellulose induced gene expression profile changes in the bacterial slurry, including an increase in bacterial flagellin expression. When administered to mice by gavage, these slurries with emulsifier‐altered expression profiles induced low‐grade inflammation and metabolic syndrome, whereas a similar slurry not treated with emulsifiers did not (Chassaing *et al.* 2017). However, to date these effects have not been confirmed in humans. Comparison with estimated dietary exposure to polysorbate 80 and carboxymethylcellulose suggests that researchers conducting these animal studies have used far higher levels of exposure than would typically be seen for the US population (Shah *et al.* 2017; Vo *et al.* 2019). Using maximum‐use levels obtained from publicly available sources, it has been estimated that lecithin and mono‐ and diglycerides have the highest mean exposures among consumers (between 60 and 80 mg/kg bodyweight/day), whereas the exposure to carboxymethylcellulose is half to one‐third less, and the exposure to polysorbate 80 is approximately half that of carboxymethylcellulose, with no additional evidence available to suggest that levels have increased since 2010 (Shah *et al.* 2017).

Emulsifiers, or surfactants/detergents, are also useful in reducing surface tension between two different substances and hence are commonly used as dishwashing detergents. It is highly plausible that contamination of food by washing detergents that have not been fully rinsed from cutlery and crockery could also be harmful if ingested (Roberts *et al.* 2013; Rhodes 2018). These detergents are often used in shampoos and toothpaste, including sodium dodecyl (lauryl) sulphate. The potential harm of washing detergents is seen in one early study which reported that dogs given regular intravenous injections of the non‐ionic detergent Triton WR‐1339 over 4‐5 months all died, with evidence of early atheroma (Scanu *et al.* 1961). A less drastic study showed that rodents ingesting washing detergent, at low levels that could plausibly be ingested by human infants, had increased permeability and irreversible atrophy of the intestinal villi (Mercurius‐Taylor *et al.* 1984).

The most extensively consumed emulsifier is the phospholipid lecithin, a natural zwitterionic surfactant present in all plant and animal cell walls (Kinyanjui *et al.* 2003). It is typically commercially sourced from soybeans and sunflowers (an alternative source increasingly used in industry as it does not need to be avoided by people with a soya allergy), but perhaps best known as a key component of egg yolks, accounting for their emulsifier properties used to make foodstuffs such as mayonnaise. Daily intake of lecithin from food sources in a typical Western diet averages about 3.6 g/day but can be up to 7 g/day, with a single egg yolk typically containing around 1.8 g of lecithin (Canty & Zeisel 1994; Palacios & Wang 2005). In contrast, total polysorbate intake is only around 10‐100 mg/day, although synthetic detergents are more resistant to breakdown by digestion (Singh *et al.* 2009). Lecithin contains varying amounts of phospholipids: phosphatidylcholine (egg lecithin 80% w/w; soy lecithin 20‐30%); phosphatidylethanolamine (egg lecithin 12%; soy lecithin 20‐30%); and phosphatidylinositol (egg lecithin 5%; soy lecithin 20%) (Palacios & Wang 2005; American Lecithin Company 2009). There are, as yet, no published studies of the impact of lecithin on either the bacterial translocation or the microbiota. Also, a considerable quantity of lecithin enters the human intestine in bile (1.4‐8.1 g/l), which, with bile secretion at around 0.75 l/day, amounts to ~ 1‐6 g/day of phosphatidylcholine entering the human intestine daily (Boyer 2013). The presence of phosphatidylcholine in bile is beneficial, helping to prevent cholesterol gallstone formation and reduce toxicity of bile acid micelles (Tompkins *et al.* 1970).

Ingestion of lecithin at high dosage in healthy human volunteers (22‐83 g/day for 2‐4 months) has shown no obvious ill effects, though a lowering of plasma triglyceride levels has been reported (Cobb *et al.* 1980). Therefore, it may have been considered unnecessary to define a safe limit, although the European Food Safety Authority (EFSA) does propose an ‘adequate intake’ of dietary choline (a quaternary amine, mainly present in lecithin as phosphatidylcholine and released during digestion by intestinal lipases) as 400 mg/day for adults (JECFA 1974a; EFSA NDA Panel 2016). Indeed, egg yolk, soy lecithin and lecithin components such as phosphatidylinositol have been shown in short‐term dietary supplementation studies to induce potentially beneficial elevations in high‐density lipoprotein (HDL)‐cholesterol (Burgess *et al.* 2005; Blesso *et al.* 2013) and a reduction in serum low‐density lipoprotein (LDL)‐cholesterol (Mourad *et al.* 2010), and encapsulated phosphatidylcholine designed for colonic delivery has shown promise in treatment of ulcerative colitis (Karner *et al.* 2014). However, dietary lecithin, or more specifically phosphatidylcholine, has been indicated as a possible risk factor for coronary artery disease, likely a consequence of its conversion of choline by the intestinal microbiota to the pro‐atherogenic metabolite trimethylamine‐N‐oxide (Tang *et al.* 2013).

The other widely consumed group of food emulsifiers is the mono‐ and diglycerides of free fatty acids (Moonen & Bas 2004). Commercially, these are semi‐synthetic, largely manufactured by enzymatic hydrolysis of triglycerides although they are also thought to occur naturally by the hydrolysis of triglycerides by lipase. There are no published data yet on their interactions with the mammalian microbiota or intestinal epithelium.

The health effects of conjugated bile acids, which are another variety of powerful detergents/emulsifiers that our intestines are continuously exposed to on a daily basis, should also be considered. We have speculated that detergents, such as bile acids, may cause harm only if they co‐exist with bacteria particularly in the terminal small intestine where, unlike the colon, there is no continuous mucus barrier (Johansson *et al.* 2014) and where, even in healthy individuals, there is substantial backwash of bacteria through the ileocaecal valve (Vince *et al.* 1972; Simon & Gorbach 1984), with a consequent mucosal colonisation that is more marked in Crohn’s disease (Gevers *et al.* 2014). The highly effective ileal reabsorption of bile acids under healthy conditions, which starts to occur at least 100 cm proximal to the ileocaecal valve (Ung *et al.* 2002), may mean that relatively little conjugated bile acid remains in the gut lumen by the distal 20 cm or so of the ileum. Conjugated bile acids, like other detergents and emulsifiers, can form micelles and thus facilitate fat absorption but also like other detergents, have a potential for cell toxicity (Raimondi *et al.* 2008). Dihydroxy bile acids (*e.g.* chenodeoxycholic and deoxycholic acid), formed by microbial dehydroxylation (*i.e*. loss of the 7α‐hydroxyl group on the bile salt nucleus), are known to enhance permeability and uptake of bacteria across the human colonic mucosa (Münch *et al.* 2007; Münch *et al.* 2011).

It has long been known that bacterial translocation from the intestine into the blood occurs in very sick individuals (*e.g.* sepsis patients in intensive care) (Quigley 2011), but it is becoming apparent that this may be much more common and particularly relevant to the pathogenesis of a number of diseases. For example, significant increases in circulating bacterial DNA have been reported in venous samples from patients with cardiovascular disease (Dinakaran *et al.* 2014), type 2 diabetes (Sato *et al.* 2014) and Crohn’s disease (Gutiérrez *et al.* 2014). In Crohn’s disease, the presence of circulating bacterial DNA has also been shown to be highly predictive of subsequent relapse (Gutiérrez *et al.* 2016). The mouse studies examining the effect of ingestion of emulsifiers polysorbate 80 and carboxymethylcellulose (Chassaing *et al.* 2015), although not directly assessing bacterial translocation across the intestine into the circulation, did report an increase in circulating anti‐lipopolysaccharide and anti‐flagellin antibody in mice consuming emulsifiers, suggesting an altered intestinal permeability and an increased exposure to bacteria‐derived molecules. Further studies by the same group showed that these emulsifiers did affect the microbiota (Chassaing *et al.* 2017), and changes in the composition of the microbiota can lead to increased bacterial translocation, as has been shown dramatically with antibiotics (Knoop *et al.* 2016). Dietary exposure to the natural emulsifier lecithin in the human diet is far higher than that seen for either polysorbate 80 or carboxymethylcellulose (Shah *et al.* 2017). Our own preliminary study in mice has suggested that ingestion of 0.1% w/v egg lecithin for 4 days in their drinking water can enhance bacterial translocation to the systemic circulation and distant organs, with levels of total bacteria measured being much greater than those we observed in mice ingesting 0.1% v/v polysorbate 80 (unpublished observations). Further evidence in mice suggests that dietary soya lecithin can enhance acute fatty acid absorption across the intestine (Couedelo *et al.* 2015) and induce inflammation and hypertrophy of white adipose tissue (Lecomte *et al.* 2016). Adipose tissue inflammation can occur as a result of enhanced lipopolysaccharide translocation across the intestine (Kim *et al.* 2012). Whether lecithin consumption in humans increases systemic levels of bacterial DNA is yet to be determined.

## Objectives of the MECNUT Emulsifier project and the FADiets study

The *Mechanistic Nutrition in Health* (*MECNUT*) *Emulsifier* project, incorporating the human *Food Additives – do processed Diets impact on gut and metabolic health* (*FADiets*) study, is funded by the Medical Research Council (MRC) (from November 2018 to October 2020) to answer two main questions:

- What impact do different emulsifiers have on the mucosal barrier, particularly in respect to bacterial translocation and inflammation?
- Does ingestion of the dietary emulsifier lecithin in controlled diets induce bacterial translocation and affect selected biomarkers of gut and metabolic health in healthy volunteers?

**Figure 3:**
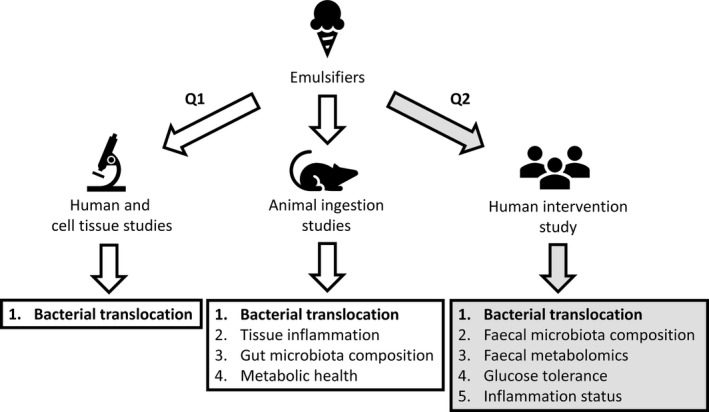
Overview of the approach to study the effects of emulsifiers on bacterial translocation, intestinal inflammation and metabolic health. We propose to use human intestinal cell cultures, 3‐D ‘mini‐gut’ organoid cultures and human ileal tissue explants, and mouse models to investigate Question (Q) 1: ‘What impact do different food emulsifiers have on the mucosal barrier particularly in respect to bacterial translocation and inflammation?’ A human volunteer study has been designed to answer Q2: ‘Does ingestion of the dietary emulsifier lecithin in controlled diets induce bacterial translocation and affect selected biomarkers of gut and metabolic health in healthy volunteers?’

An overview of the proposed approach is presented in Figure 3.

### Objective 1: To assess the impact of a wide range of commercially‐used food emulsifiers, dihydroxyl bile salts (as a source of natural detergents) and synthetic dish washing detergents on the mucosal barrier, particularly in respect of bacterial translocation and inflammation

The *MECNUT Emulsifier* study will implement a three‐step model approach, examining the effect of treatment or ingestion of a wide range of permitted food emulsifiers, bile salts and household dishwashing detergents (see Table 2), using the following:

- *in vitro* human cell lines of the intestinal epithelium, an M‐cell model of the follicle‐associated epithelium and intestinal crypt stem‐cell derived 3‐dimensional (3‐D) ‘mini‐gut’ organoid cultures;
- *ex vivo* human distal ileum tissue explants mounted in Ussing chambers; and
- *in vivo* mouse studies.

#### In vitro cell‐line studies

Expanding on our previous research (Roberts *et al.* 2010), to explore how a wide range of emulsifiers influence bacterial translocation across the intestinal epithelium, we will use three well‐characterised human intestinal epithelial cell‐line cultures: fully differentiated Caco2 (modelling small intestinal enterocytes), HT29 (colonocytes) (Martin *et al.* 2004) and Caco2‐Raji B lymphocyte co‐cultures [an M‐cell model of the follicle‐associated epithelium (Roberts *et al.* 2010)]. These will be grown as monolayers on Millicell™ membrane culture plate inserts to allow treatment and sampling of the apical and basolateral aspects. As a primary endpoint, the ability of emulsifiers to affect transcellular movement of bacteria (*i.e.* entry through cells within the monolayer) will be monitored over 4 hours. Emulsifiers will be added apically to cell monolayers for 30 minutes prior to addition of *E. coli* or *Salmonella,* along with low molecular weight (3 kDa) dextran to monitor paracellular permeability (*i.e*. ‘leakiness’ of cell–cell tight junctions). Transepithelial electrical resistance will also be monitored to provide additional information about the integrity of the tight junctions formed between the polarised cells of the monolayers throughout all treatment stages. Emulsifiers and detergents will be used at concentration ranges found within a typical Western diet, as well as below and up to those that would start to disrupt cell–cell tight junctions (Münch *et al.* 2007; Roberts *et al.* 2010; Münch *et al.* 2011). For polysorbates, such as polysorbate 80, known to show resistance to digestion (Singh *et al.* 2009), we previously calculated that 0.01% v/v would be realistic (Roberts *et al.* 2010). Based on the acceptable daily intake of 25 mg/kg bodyweight (JECFA 1974b), a level of 0.01% would represent a persistence of 6.7% into the terminal ileum of a typical 60 kg human, assuming 1l of intestinal contents per day passing to the caecum. Assuming 1 g/ml [approximately correct for lecithin based on daily output of biliary phosphatidyl choline plus daily dietary intake of lecithin (JECFA 1974a; Boyer 2013)] then for lecithin, this would be ~ 6% v/v entering the caecum assuming 1 l/day of intestinal contents entering caecum – but this would of course allow for no breakdown of lecithin during digestion (Szuhaj 1989), so study levels up to 5% w/v likely would be appropriate. For dishwashing detergents, Mercurius‐Taylor and colleagues calculated intake in adults of ~ 1 mg/kg/day arising from residue left on detergent washed (5 ml detergent in 2 l tap water), unrinsed crockery and glassware (Mercurius‐Taylor *et al.* 1984) – again assuming 1 l/day entering caecum and no breakdown or absorption proximally, this would amount to 7 mg/100 ml (*i.e.* 0.007% v/v). For bile salts, levels to be tested are as defined by Münch *et al. *(2007, 2011).

Study of some of the most commercially used non‐ionic series of emulsifiers [such as the 6 fatty acid ester series of sorbitan (Span 20 to 85) and their ethoxylated derivatives (polysorbates 20 to 85)] may enable us to correlate the effect of the head group and hydrophobic tail of the surfactants with their functional behaviour (Tadros 2005). We have already shown differences in the ability of polysorbate 60 and polysorbate 80 to promote bacterial translocation through intestinal epithelial cells and M cells in culture without impacting on membrane paracellular permeability (*i.e.* a transcytotic effect), with polysorbate 60 showing less marked ability to effect translocation (Roberts *et al.* 2010).

Key emulsifiers/detergents identified as enhancing epithelial transcytosis of bacteria in the human cell‐line studies will be further studied using ‘mini‐gut’ organoid cultures. Generated from intestinal crypt tissue stem cells, organoids (both those from the small and large intestine) mimic the complex 3D architecture of the epithelium, with all differentiated cell types being present (Nigro *et al. * ). Emulsifiers will be microinjected along with enhanced green fluorescent protein (EGFP)‐expressing *E. coli* and a paracellular permeability dye to monitor organoid epithelium integrity. These ‘mini‐gut’ models will serve to bridge the *in vitro* and *in vivo* work. Experiments will be carried out using emulsifiers both in solution (where possible) and as emulsions, in combination with low and high‐fat levels, to resemble more closely food structures as influenced by process and ingredient interactions, and interactions taking place within the intestinal lumen.

#### Ex vivo human tissue studies

Emulsifiers/detergents identified as affecting translocation of bacteria in the *in vitro* and *ex vivo* models, also including egg and soy lecithin, and polysorbate 80 as comparators (Roberts *et al.* 2010), will be assayed for their ability to enhance translocation of EGFP‐expressing *E. coli* and/or *Salmonella* across villous epithelium isolated from fresh, macro/microscopically normal, human distal ileum tissue removed during routine surgery (*e.g.* for colon cancer) and mounted in Ussing chambers as previously described (Roberts *et al.* 2010; Chassaing *et al.* 2011). Again, transepithelial electrical resistance will be monitored to assess any changes in paracellular permeability.

#### In vivo mouse studies

Impact of ingestion (either in drinking water or incorporated within the diet, short‐ and long‐term) of five emulsifiers [polysorbate 80, lecithin and sodium dodecyl (lauryl) sulphate plus two selected from the *in vitro* studies] will be studied in wild‐type C57BL/6J and *Il‐10^‐/‐^* mice (a colitis‐susceptible model relevant to human IBD). The primary endpoint will be detection of venous blood bacterial DNA as a marker of microbiota translocation from the intestinal lumen to the circulation and also to the systemic organs such as the liver, spleen and kidneys. Evidence of altered intestinal barrier function (Williams *et al.* 2013), histological intestinal inflammation, bacterial DNA in venous blood, systemic organs and community alterations in the intestinal microbiota [using total bacteria, phyla‐ and class‐specific qPCR (Bacchetti De Gregoris *et al.* 2011)] will also be evaluated.

#### Objective 2: To assess whether ingestion of the dietary emulsifier lecithin in controlled diets induces bacterial translocation and affects selected biomarkers of gut and metabolic health in healthy volunteers

The *FADiets* study primarily aims to determine whether short‐term (2 week), high intake of lecithin alters gut function, as indicated by the increased presence of bacterial DNA in the circulation (venous blood sampling), increased gut inflammation (faecal sampling for white blood cell components such as calprotectin), gut microbiota and metabolic activity [bacterial diversity, faecal short‐chain fatty acids (SCFAs) and volatile organic compounds] and altered glucose metabolism (assessed by oral glucose tolerance test).

**Figure 4:**
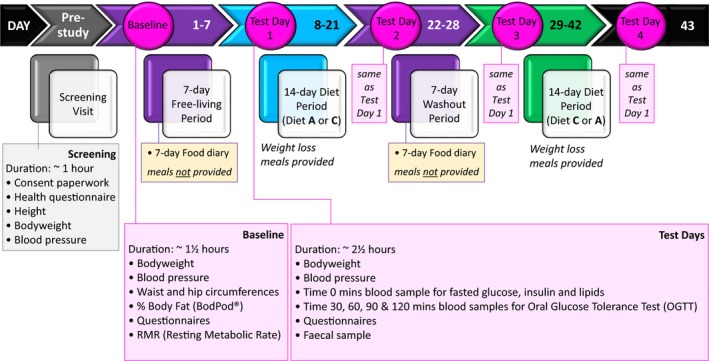
The FADiets study protocol summary. The research question: ‘Does dietary intake of soy lecithin alter the intestinal lining and the microbes that normally exist in the intestinal lumen, in healthy subjects, consumed over a 2‐week period (in comparison to a low‐emulsifier diet)?’ [Colour figure can be viewed at http://www.wileyonlinelibrary.com/]

The study will implement a 5‐week randomised crossover dietary intervention trial (https://clinicaltrials.gov/ Identifier: NCT03842514), in overweight or obese (but otherwise healthy) adults (BMI ranging from 27 to 40 kg/m^2^), comparing a ‘low‐calorie, low‐emulsifier’ diet, with a ‘low‐calorie, high‐emulsifier’ diet (Fig. 4). This population was chosen to increase recruitment and retainment of subjects throughout the study by offering the opportunity to lose weight should they follow our diets. Both diets are weight loss (low‐calorie) diets, fed to basal energy requirements with below average amounts of red meat, fish and eggs [compared with amounts consumed by the UK population according to *National Diet and Nutrition Survey* data (Bates *et al.* 2014)] to ensure a relatively low lecithin intake, so participants are likely therefore to be more compliant and consume all the food provided. Low‐calorie diets provided are identical in all aspects (energy, macronutrients and foods) so as to counter any possible microbiota effects driven by the energy content of the diet, except for the addition of 15 g (2 x 7.5 g/day) soya lecithin to the high‐emulsifier diet. The low‐emulsifier diet consisting of commercially available foods (of verified ingredient composition) will provide ~ 0.3 g/day choline [which is below the EFSA adequate daily intake (ADI) and US Department of Agriculture (USDA) guidance minimum for adults of 0.4 g/day (EFSA NDA Panel 2016; USDA 2019)] from food, and the emulsifier supplemented diet will provide 3.7 g/day choline (1.73 g choline twice daily in the form of the soya lecithin granules – which is 3.46 g/day), giving 3.7 g in total from food and the supplement (Table 4).

**Table 4:** Low‐calorie intervention diets provided to volunteers on the FADiets study

**Low calorie, high emulsifier diet** *					
**Day**	**Energy** **†** ** (kcal)**	**Fat (%)**	**Protein (%)**	**Carbohydrates/fibre (%)**	**Choline** **‡** ** (from diet & lecithin supplement) (mg)**
1	2000	30	15	55	3717.4
2	2000	30	15	55	3698.2
3	2000	30	15	55	3810.1
4	2000	30	15	55	3707.7
5	2000	30	15	55	3783.2
6	2000	30	15	55	3722.7
7	2000	30	15	55	3725.7
Average					3737.9
**Low calorie, low emulsifier diet**					
**Day**	**Energy** **†** ** (kcal)**	**Fat (%)**	**Protein (%)**	**Carbohydrates/fibre (%)**	**Choline** **‡** ** (from diet only) (mg)**
1	2000	30	15	55	270.4
2	2000	30	15	55	263.1
3	2000	30	15	55	367.1
4	2000	30	15	55	261.6
5	2000	30	15	55	339.9
6	2000	30	15	55	273.1
7	2000	30	15	55	265.6
Average					291.5

* Low‐calorie test diet supplemented with 2 daily servings of 7.5 g Lamberts® soya lecithin granules in fruit smoothies (total 15 g/day).

^†^ Energy intakes matched to the closest calorie to each volunteer resting metabolic rate assessed at baseline by indirect calorimetry (range 1500 to 3000 kcal). Example 2000 kcal matched diets are shown.

^‡^ Choline values from USDA food composition tables (USDA, 2019). EFSA Adequate Daily Intake (ADI) of choline, 400 mg/day for adults (EFSA NDA Panel 2016) and USDA ADI, male 550 mg/day and female 425mg/day (USDA 2019).

The dietary intervention will consist of two 14‐day diet periods, with either no added emulsifier or 15 g soya lecithin per day, with a 7‐day baseline habitual diet (‘free‐living’) period [*i.e.* to reflect normal eating patterns of participants] and a 7‐day mid‐study washout [*i.e.* a return to normal (habitual) diet], so that both periods prior to intervention are the same (habitual food intake will be recorded using food diaries). A high‐profile human dietary intervention study (David *et al.* 2014) used a similar period of washout to ensure recovery of microbial diversity following each study arm examined, and we have also previously shown that the microbiota responds quickly to changes in diet and that these changes are rapidly reversed by intervention with a subsequent diet (Walker *et al.* 2011). All food and drinks for the intervention diets will be prepared by the Human Nutrition Unit at the Rowett Institute for volunteers to collect, reheat and consume at home. Volunteers will undergo initial health screening and confirmation of eligibility to participate and will be randomised for treatment order, to be conducted by computer generation by our statistical support at Biomathematics and Statistics Scotland. All volunteers will be matched to the closest calorie to their resting metabolic rate (assessed at baseline by indirect calorimetry). The study will be conducted in a non‐blinded method with diets colour‐coded.

The lecithin supplement to be used is a commercial source of soya lecithin granules [Lamberts® – a food‐grade product, manufactured to the stringent pharmaceutical standards of Good Manufacturing Practice (GMP); composition summarised in Table 5]. It will be ingested twice daily (at a dose of 7.5 g), incorporated into a fruit smoothie matrix for stability, consistency of preparation and consumer acceptability. The total dose (15 g/day) is substantially greater than the typical dietary intake of up to 7 g/day, so is expected to be enough to test for possible effects in what is a relatively short‐term study. Previous work has reported that 7.5 g of lecithin, ingested three times daily for 4 weeks and had no adverse effects in human volunteers (Cobb *et al.* 1980), but this study did not measure bacterial translocation or changes to the gut microbiota.

**Table 5:** Fat and phospholipid composition of Lamberts® soya lecithin granules used to supplement the FADiets study low‐calorie, high‐emulsifier test diet

Component	Amount (g) per 7.5 g serving*
Phosphatidyl choline	1.7
Phosphatidyl ethanolamine	1.5
Phosphatidyl inositol	1.1
Phosphatidic acid	0.6
Phosphatidyl serine	0.075
Fatty acids	3.8
of which: – saturated	0.9
– monounsaturated	0.3
– polyunsaturated	2.5

Full composition can be found at http://www.lambertshealthcare.co.uk

* Participants on low‐calorie, high‐emulsifier diet will ingest within food a 7.5 g serving twice daily.

All study participants will keep a daily weighed food intake diary during the 7‐day baseline and 7‐day washout periods, and complete a gastrointestinal discomfort questionnaire (Storey *et al.* 2007). Stool samples will be collected during both arms and during the washout period, with faecal 16S rRNA gene sequencing and volatile organic compound analysis (measuring alterations in microbiota genes and metabolic activity, respectively) performed on all available samples.

The primary objective will be to assess bacterial translocation in response to a diet containing soya lecithin emulsifier, in comparison with the control period of a low‐emulsifier diet. Bacterial DNA in venous blood sampled at the start and end of each diet period will be measured by qPCR (Bacchetti De Gregoris *et al.* 2011). In support, serum lipopolysaccharide‐binding protein and soluble CD14 will also be measured as secondary markers of bacterial translocation to the circulation (Landmann *et al.* 1995; Blairon *et al.* 2003; Knapp *et al.* 2003; Uhde *et al.* 2016).

Secondary endpoints of the *FADiets* study include changes in bacterial diversity and metabolic activity, gut inflammation and glucose metabolism. Alterations to the composition of the gut microbiota have been observed in animal models following exposure to emulsifiers (Table 3), with some reporting increased *Proteobacteria* (Chassaing *et al.* 2015; Furuhashi *et al.* 2019). We will monitor for any differences in faecal bacterial community structure between habitual diet, and the high‐ and low‐emulsifier diets by Illumina MiSeq sequencing of the V1–V2 regions of bacterial 16S rRNA genes isolated from participant faecal samples. Taxonomic profiles as well as microbiota diversity measures such as Shannon and inverse Simpson indices will be made as previously described (Chung *et al.* 2016; Reichardt *et al.* 2018). Short‐chain fatty acids, such as acetate, butyrate and propionate, generated by the majority of bacteria in the intestine, are a major fuel source for epithelial cells lining the bowel (Reichardt *et al.* 2018), and some, such as butyrate, may have anti‐inflammatory and anti‐carcinogenic effects (Hamer *et al.* 2008). Levels of SCFAs are therefore indicative of bowel health, and participant faecal samples will be analysed for key SCFAs by gas chromatography profiling (Richardson *et al.* 1989). Volatile organic compounds present in faeces, resulting from the combined metabolism of the gut mucosa and microbiota, can be indicative of intestinal infection and inflammation, so participant sample volatile organic compounds will be measured using optimised methods for analysis of faecal headspace gases by gas chromatography‐mass spectrometry (Ahmed *et al.* 2016). Faecal calprotectin levels will also be monitored as these have been found to be significantly increased in stools of patients with IBD, whereas they are not elevated in patients with non‐inflammatory functional diseases such as irritable bowel syndrome (Burri & Beglinger 2014). Plasma trimethylamine‐N‐oxide, as a measure of cardiovascular risk (Tang *et al.* 2013), will also be monitored. Plasma fasting glucose and glucose tolerance will be assessed in study participants using a standard 75 g 2‐hour oral glucose tolerance test at the start and end of each dietary intervention period. In addition, circulating lipids and levels of insulin will be measured, with appropriate calculations performed to estimate insulin sensitivity (Blesso *et al.* 2013).

### Proposed mechanism of action

**Figure 5:**
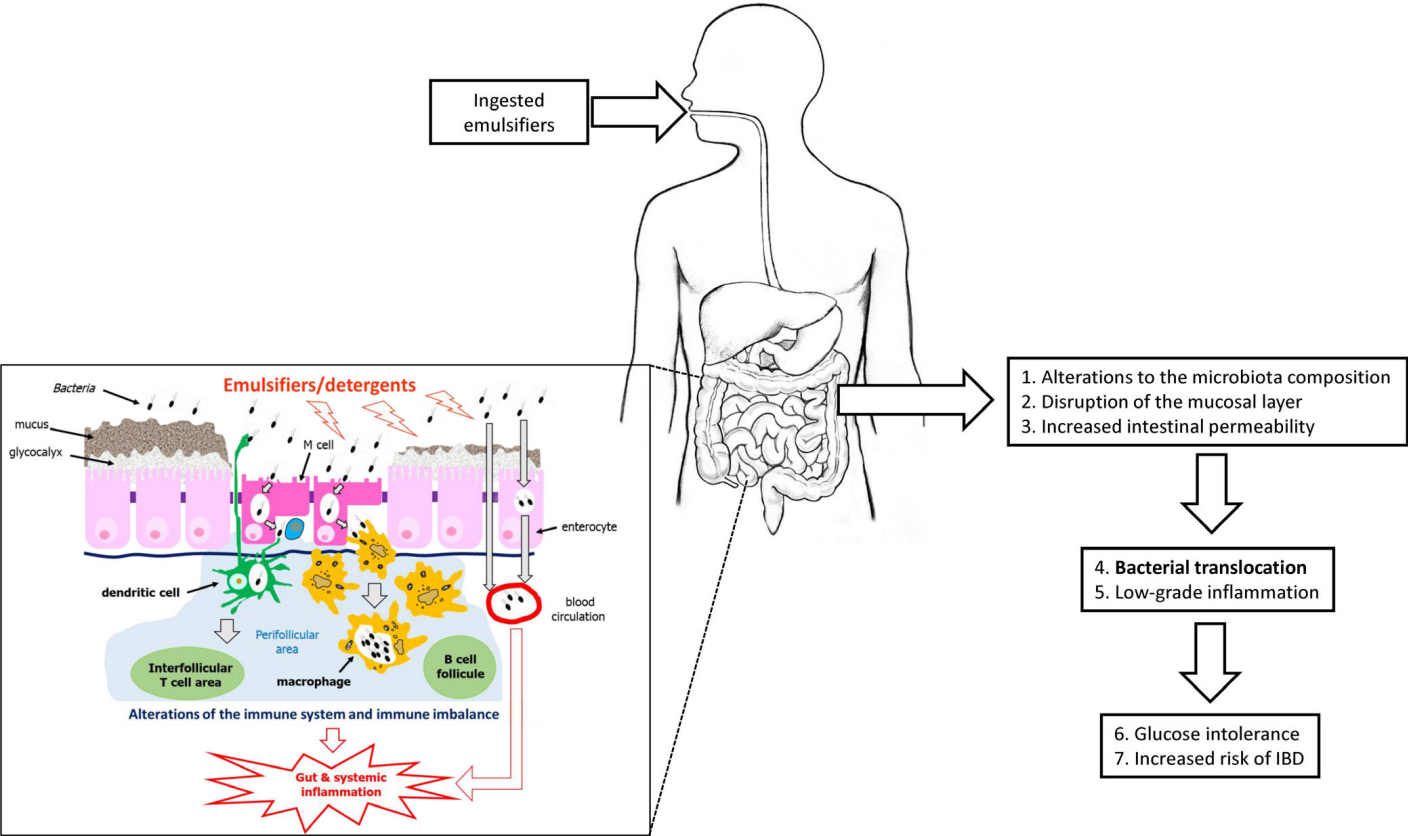
Overview of proposed adverse health effects of ingested emulsifiers. The insert shows the hypothesised mechanisms of action of emulsifiers on altering the microbiota, causing disruption of the mucosal barrier, enhancing bacterial translocation through and between intestinal epithelial cells and increasing uptake across M (microfold)‐cells of the follicle‐associated epithelium. This may lead to immune imbalance, increased levels of bacteria in the circulation and increased gut and systemic inflammation. IBD, inflammatory bowel disease [Colour figure can be viewed at http://www.wileyonlinelibrary.com/]

Our proposed mechanism of action for some commonly used dietary emulsifiers, consumed daily at low levels, on gut and metabolic health is that they may (1) cause alterations to the gut microbiota and (2) disrupt the intestinal mucosal barrier. Together, these (3) contribute to increased permeability of the intestinal layer and (4) promote the increased translocation of bacteria from the gut to the bloodstream. This results in (5) a state of low‐grade inflammation, (6) glucose intolerance and (7) increased risk of IBD (Fig. 5). Within the series of experiments proposed above, we will study all the proposed constituents, apart from the end result of (7) increased risk of IBD, due to the chronic nature of this outcome.

## Beneficiaries and public health message

The expectation is that the results of these proposed studies will lead to greater public awareness and reduction where need is identified, in the use of certain emulsifiers in the food chain that have potential to cause harm. The academic nutrition and public health communities, with the scientific advice of key independent food safety authorities (*e.g.* EFSA and JECFA, the Joint Food and Agriculture Organization/World Health Organization Expert Committee on Food Additives) examining food additives, food processing and changing consumption patterns across populations, may then be better placed to support informed choice for healthier diet planning, not only to better develop during infancy and enjoy a healthy old age, but also including support towards dietary strategies for combating IBD, cardiovascular risk and metabolic disorders, such as metabolic syndrome, type 2 diabetes and obesity.

The data obtained would be of interest to those working in basic scientific fields of nutrition and intestinal biology, and those working in the fields of gut ‘omics’ and gut health. Outcomes would also be of significant interest to formulation scientists working in and around the food industry (*e.g*. in food composition, surfactant and natural biopolymer formulation science), at leading companies supplying the food, nutrition and pharmaceutical (drug formulation and delivery) emulsifier market, and to key providers of further education in agri‐food science, food industry development, technologies and practice. As an example, the Food Additives and Ingredients Association works with both the food industry and consumers, to promote a better understanding of the role of food additives and functional food ingredients in a healthy and safe diet.

## Dissemination

Overall, the outcomes of this research proposal will add to the literature on whether excessive exposure to emulsifiers in the food chain could be harmful to health; data relevant to the key international health and food safety regulatory authorities, the scientific food research community and the food industry (both processing and additive formulation).

## Conclusions

Emerging *in vitro* and animal evidence suggests that food additives such as emulsifiers may contribute to gut and metabolic disease development through alterations to the gut microbiota, intestinal mucus layer, increased bacterial translocation and associated inflammatory response. The *MECNUT Emulsifier* project aims to further explore the mechanistic basis for these relationships across a wide range of permitted dietary emulsifiers and detergents *in vitro*. As part of this work package, the *FADiets* study aims to determine the impact of soy lecithin on gut and metabolic health *in vivo* using a controlled dietary intervention. This growing area of nutritional science may lead to innovative knowledge, which could pave new ways of addressing gut and metabolic health via implementing dietary guidelines directed at food additives.

## Conflicts of interest

No conflicts of interest have been declared by any author.

## Funding

This study is funded by the Medical Research Council (MR/P023606/1).

## Ethics

The human volunteer *FADiets* study (https://clinicaltrials.gov/ Identifier: NCT03842514) will be conducted in compliance with the ‘principles of Good Clinical Practice’ for non‐CTIMP/MHRA studies. In addition to Sponsorship approval, ethical approval has been obtained from the Rowett Institute Ethics Committee (approval date 11/02/2019, internal study number 810). *Ex vivo* studies using freshly resected macro/microscopically human ileum tissue removed during routine surgery (*e.g.* for colon cancer) will be obtained following informed written consent and with approval of the Regional Human Ethics Committee; Linköping, Sweden.

## Author Contributions

All authors contributed to the writing and preparation of the manuscript.
